# Supplementary material for: Prognostic value of the systemic immune-inflammation index in lung cancer patients receiving immune checkpoint inhibitors: A meta-analysis
Source: PLoS One. 2024 Nov 1;19(11):e0312605. doi: 10.1371/journal.pone.0312605 (PMC11530041; doi:10.1371/journal.pone.0312605)
Supplement: S1 Table — (DOCX) [file pone.0312605.s002.docx]

Supplementary Table 1. Information about 179 records from databases and reasons of their inclusion or exclusion.

| Publications | Included or excluded with corresponding reasons |
| --- | --- |
| 1. Mantovani A, Allavena P, Sica A, Balkwill F. Cancer-related inflammation. Nature 2008; 454(7203): 436-44.  2. 李仁科. 七氟醚和丙泊酚单肺通气麻醉对应激的影响 [硕士]: 郑州大学; 2009.  3. Diakos CI, Charles KA, McMillan DC, Clarke SJ. Cancer-related inflammation and treatment effectiveness. The Lancet Oncology 2014; 15(11): e493-503.  4. Diakos CI, Charles KA, McMillan DC, Clarke SJ. Cancer-related inflammation and treatment effectiveness. The Lancet Oncology 2014; 15(11): e493-503.  5. Borghaei H, Paz-Ares L, Horn L, et al. Nivolumab versus Docetaxel in Advanced Nonsquamous Non-Small-Cell Lung Cancer. The New England journal of medicine 2015; 373(17): 1627-39.  6. Gettinger SN, Horn L, Gandhi L, et al. Overall Survival and Long-Term Safety of Nivolumab (Anti-Programmed Death 1 Antibody, BMS-936558, ONO-4538) in Patients With Previously Treated Advanced Non-Small-Cell Lung Cancer. Journal of clinical oncology : official journal of the American Society of Clinical Oncology 2015; 33(18): 2004-12.  7. 陈晶. TLR9激动剂CpG-ODN对小鼠放射肺损伤的免疫影响及机制研究 [博士]: 武汉大学; 2016.  8. Buchbinder EI, Desai A. CTLA-4 and PD-1 Pathways: Similarities, Differences, and Implications of Their Inhibition. American j*ournal of clinical oncology* 2016; **39**(1): 98-106.  9. Diem S, Schmid S, Krapf M, et al. Neutrophil-to-Lymphocyte ratio (NLR) and Platelet-to-Lymphocyte ratio (PLR) as prognostic markers in patients with non-small cell lung cancer (NSCLC) treated with nivolumab. *Lung Cancer* 2017; **111**: 176-81.  10. 张馨元. RIPC对单肺通气老年患者脑氧代谢和术后早期认知功能的影响 [硕士]: 昆明医科大学; 2018.  11. Akamine T, Takada K, Toyokawa G, et al. Association of preoperative serum CRP with PD-L1 expression in 508 patients with non-small cell lung cancer: A comprehensive analysis of systemic inflammatory markers. *Surgical Oncology* 2018; **27**(1).  12. Chovanec M, Cierna Z, Miskovska V, et al. Systemic immune-inflammation index in germ-cell tumours. *British Journal of Cancer* 2018; **118**(6): 831-8.  13. Akamine T, Takada K, Toyokawa G, et al. Association of preoperative serum CRP with PD-L1 expression in 508 patients with non-small cell lung cancer: A comprehensive analysis of systemic inflammatory markers. *Surgical Oncology* 2018; **27**(1).  14. Suh KJ, Kim SH, Kim YJ, et al. Post-treatment neutrophil-to-lymphocyte ratio at week 6 is prognostic in patients with advanced non-small cell lung cancers treated with anti-PD-1 antibody. *Cancer immunology, immunotherapy : CII* 2018; **67**(3): 459-70.  15. 齐秋晨. 中药益肺通络方及其改良方对肺癌生长和转移的抑制作用及其机制研究 [博士]: 山东大学; 2019.  16. 张化杰. 疾病相关糖抗原和糖抗体的制备、分析及免疫学应用 [博士]: 山东大学; 2019.  17. 张鑫. 肺癌患者呼吸道菌群结构及代谢组特征分析与比较研究 [博士]: 中国人民解放军海军军医大学; 2019.  18. 张秀蕾. 地钱素C和内质网蛋白RCN1对肺癌细胞衰老及相关分泌表型的调控作用研究 [博士]: 山东大学; 2019.  19. Rossana B, Matteo S, Silvia R, et al. Pre-treatment systemic immune-inflammation represents a prognostic factor in patients with advanced non-small cell lung cancer. *Annals of translational medicine* 2019; **7**(20).  20. Sofia A, Konstantinos R, Chara P, et al. Cancer cachexia, sarcopenia and hand-GRIP strength (HGS) in the prediction of outcome in patients with metastatic non-small cell lung cancer (NSCLC) treated with immune checkpoint inhibitors (ICIs): A prospective, observational study. *Journal of Clinical Oncology* 2019; **37**(15_suppl).  21. Wang Y, Li Y, Chen P, Xu W, Wu Y, Che G. Prognostic value of the pretreatment systemic immune-inflammation index (SII) in patients with non-small cell lung cancer: a meta-analysis. *Annals of Translational Medicine* 2019; **7**(18).  22. Wang Y, Li Y, Chen P, Xu W, Wu Y, Che G. Prognostic value of the pretreatment systemic immune-inflammation index (SII) in patients with non-small cell lung cancer: a meta-analysis. *Ann Transl Med* 2019; **7**(18): 433.  23. Xinyue W, Lianjing C, Shouying L, Fan W, Dingzhi H, Richeng J. Combination of PD-L1 expression and NLR as prognostic marker in patients with surgically resected non-small cell lung cancer. *Journal of Cancer* 2019; **10**(26).  24. Zhang Y, Chen B, Wang L, Wang R, Yang X. Systemic immune-inflammation index is a promising noninvasive marker to predict survival of lung cancer: A meta-analysis. *Medicine* 2019; **98**(3): e13788.  25. 董美莲. CYTL1与系统免疫炎症指数预测局部晚期宫颈鳞癌同步放化疗预后的研究 [博士]: 郑州大学; 2020.  26. 江红. RIPC联合乌司他丁对单肺通气老年患者脑氧代谢和术后谵妄的影响 [硕士]: 昆明医科大学; 2020.  27. 康静静. 食管癌术后同步放化疗的作用及PD-L1表达的预后价值立体定向放疗后早期非小细胞肺癌的Nomogram模型及cfDNA对ⅢA-N2期非小细胞肺癌的预后价值 [博士]: 北京协和医学院; 2020.  28. 李步托. 基于影像组学的多维度深度学习模型预测贝伐单抗治疗晚期非小细胞肺癌患者预后的研究 [博士]: 天津医科大学; 2020.  29. 王丽芬. 微卫星不稳定在散发性结直肠癌中的表达及其与预后的相关性研究 [博士]: 山东大学; 2020.  30. 于文洁. 口腔及口咽癌系统性炎性标志物及其与微环境CD15+细胞及MDA5表达预后意义的研究 [博士]: 山东大学; 2020.  31. Alex F, Alfredo A. Promising predictors of checkpoint inhibitor response in NSCLC. *Expert review of anticancer therapy* 2020; **20**(11): 931-7.  32. Burak B, Mutlu H, Şebnem Y, et al. The association of clinicopathologic features and peripheral blood parameters with high PD-L1 expression in non-small cell lung cancer. *Tuberkuloz ve toraks* 2020; **68**(2).  33. C BJ, Gonzalo R, Elie R, Laura M. The LIPI score and inflammatory biomarkers for selection of patients with solid tumors treated with checkpoint inhibitors. *The quarterly journal of nuclear medicine and molecular imaging : official publication of the Italian Association of Nuclear Medicine (AIMN) [and] the International Association of Radiopharmacology (IAR), [and] Section of the Society of* 2020; **64**(2).  34. Burak B, Mutlu H, Şebnem Y, et al. The association of clinicopathologic features and peripheral blood parameters with high PD-L1 expression in non-small cell lung cancer. *Tuberkuloz ve toraks* 2020; **68**(2).  35. Inoue T, Ito S, Kanda M, et al. Preoperative six-minute walk distance as a predictor of postoperative complication in patients with esophageal cancer. *Diseases of the esophagus : official journal of the International Society for Diseases of the Esophagus* 2020; **33**(2).  36. Inoue T, Ito S, Kanda M, et al. Preoperative six-minute walk distance as a predictor of postoperative complication in patients with esophageal cancer. *Diseases of the esophagus : official journal of the International Society for Diseases of the Esophagus* 2020; **33**(2).  37. Killock D. TMB - a histology-agnostic predictor of the efficacy of ICIs? *Nature reviews Clinical oncology* 2020; **17**(12): 718.  38. Li Y, Zhang Z, Hu Y, et al. Pretreatment Neutrophil-to-Lymphocyte Ratio (NLR) May Predict the Outcomes of Advanced Non-small-cell Lung Cancer (NSCLC) Patients Treated With Immune Checkpoint Inhibitors (ICIs). *Frontiers in oncology* 2020; **10**: 654.  39. Li Y, Zhang Z, Hu Y, et al. Pretreatment Neutrophil-to-Lymphocyte Ratio (NLR) May Predict the Outcomes of Advanced Non-small-cell Lung Cancer (NSCLC) Patients Treated With Immune Checkpoint Inhibitors (ICIs). *Frontiers in oncology* 2020; **10**: 654.  40. M HB, S GA, Evguenia S, et al. Immune checkpoint protein VSIG4 as a biomarker of aging in murine adipose tissue. *Aging cell* 2020; **19**(10).  41. Petrova MP, Donev IS, Radanova MA, et al. Sarcopenia and high NLR are associated with the development of hyperprogressive disease after second-line pembrolizumab in patients with non-small-cell lung cancer. *Clinical and experimental immunology* 2020; **202**(3): 353-62.  42. Petrova MP, Donev IS, Radanova MA, et al. Sarcopenia and high NLR are associated with the development of hyperprogressive disease after second-line pembrolizumab in patients with non-small-cell lung cancer. *Clinical and experimental immunology* 2020; **202**(3): 353-62.  43. Petrova MP, Donev IS, Radanova MA, et al. Sarcopenia and high NLR are associated with the development of hyperprogressive disease after second-line pembrolizumab in patients with non-small-cell lung cancer. *Clinical and experimental immunology* 2020; **202**(3): 353-62.  44. Wang Y, Zhang Q, Miao L, Zhou Y. Nivolumab in combination with anlotinib achieved remarkable efficacy in a patient with driver-negative lung squamous cell carcinoma and PS of 4. *Annals of palliative medicine* 2020; **9**(6): 4384-8.  45. 陈晓博, 王倩, 李庆霞. 系统性免疫炎症指数与非小细胞肺癌患者预后关系的研究进展. *肿瘤防治研究* 2021; **48**(06): 652-6.  46. 刘杰. 免疫检查点抑制剂治疗晚期非小细胞肺癌的疗效与安全性分析 [硕士]: 西安医学院; 2021.  47. 孙一丹. “扶正解毒祛瘀法”参与治疗小细胞肺癌的回顾性研究 [硕士]: 天津中医药大学; 2021.  48. 周树波. 兰坪虫草多糖联合顺铂增强Lewis荷瘤小鼠的抗肿瘤活性研究 [硕士]: 昆明理工大学; 2021.  49. Can GD, Aral OD, Koray ST, et al. MO368THE INCIDENCE AND RISK FACTORS FOR ACUTE KIDNEY INJURY IN PATIENTS TREATED WITH IMMUNE CHECKPOINT INHIBITORS: A REAL-LIFE STUDY. *Nephrology Dialysis Transplantation* 2021; **36**(Supplement1).  50. Du F, Qiu Z, Ai W, et al. Blood tests predict the therapeutic prognosis of anti-PD-1 in advanced biliary tract cancer. *Journal of Leukocyte Biology* 2021; **110**(2): 327-34.  51. Ela DS, Yasemin K, Cengiz K, Ozden K, Mumin K, Suheyla AA. Prognostic impact of immune inflammation biomarkers in predicting survival and radiosensitivity in patients with non-small-cell lung cancer treated with chemoradiotherapy. *Journal of medical imaging and radiation oncology* 2021; **66**(1).  52. Ela DS, Yasemin K, Cengiz K, Ozden K, Mumin K, Suheyla AA. Prognostic impact of immune inflammation biomarkers in predicting survival and radiosensitivity in patients with non-small-cell lung cancer treated with chemoradiotherapy. *Journal of medical imaging and radiation oncology* 2021; **66**(1).  53. Fornarini G, Rebuzzi SE, Banna GL, et al. Immune-inflammatory biomarkers as prognostic factors for immunotherapy in pretreated advanced urinary tract cancer patients: an analysis of the Italian SAUL cohort. *ESMO open* 2021; **6**(3): 100118.  54. Fornarini G, Rebuzzi SE, Banna GL, et al. Immune-inflammatory biomarkers as prognostic factors for immunotherapy in pretreated advanced urinary tract cancer patients: an analysis of the Italian SAUL cohort. *ESMO open* 2021; **6**(3).  55. Fornarini G, Rebuzzi SE, Banna GL, et al. Immune-inflammatory biomarkers as prognostic factors for immunotherapy in pretreated advanced urinary tract cancer patients: an analysis of the Italian SAUL cohort. *ESMO open* 2021; **6**(3).  56. Grant MJ, Herbst RS, Goldberg SB. Selecting the optimal immunotherapy regimen in driver-negative metastatic NSCLC. *Nature reviews Clinical oncology* 2021; **18**(10): 625-44.  57. Kauffmann-Guerrero D, Kahnert K, Kiefl R, et al. Systemic inflammation and pro-inflammatory cytokine profile predict response to checkpoint inhibitor treatment in NSCLC: a prospective study. *Scientific reports* 2021; **11**(1).  58. M.Y. H, P. X, B.G. C, C. Z. The Prognostic Significance of Systemic Immune-Inflammation Index in Patients With Glioblastoma. *International Journal of Radiation Oncology, Biology, Physics* 2021; **111**(3S).  59. Qi W-X, Xiang Y, Zhao S, Chen J. Assessment of systematic inflammatory and nutritional indexes in extensive-stage small-cell lung cancer treated with first-line chemotherapy and atezolizumab. *Cancer Immunology, Immunotherapy* 2021; **70**(11).  60. Shan T, Yinghao C, Yanran D, Qi L, Pailan P. Gustave Roussy Immune Score as a Novel Prognostic Scoring System for Colorectal Cancer Patients: A Propensity Score Matching Analysis. *Frontiers in oncology* 2021; **11**.  61. Shan T, Yinghao C, Yanran D, Qi L, Pailan P. Gustave Roussy Immune Score as a Novel Prognostic Scoring System for Colorectal Cancer Patients: A Propensity Score Matching Analysis. *Frontiers in oncology* 2021; **11**.  62. Xiong Q, Huang Z, Xin L, et al. Post-treatment neutrophil-to-lymphocyte ratio (NLR) predicts response to anti-PD-1/PD-L1 antibody in SCLC patients at early phase. *Cancer immunology, immunotherapy : CII* 2021; **70**(3): 713-20.  63. The relationship between NLR/PLR/LMR levels and survival prognosis in patients with non-small cell lung carcinoma treated with immune checkpoint inhibitors: Erratum. *Medicine* 2022; **101**(41): e31359.  64. 艾忻. 基于真实世界的晚期肺癌疗效预测 [硕士]: 北京协和医学院; 2022.  65. 白阳. N6-甲基腺苷RNA甲基化调节模式评分在肺腺癌中预后评估及气道介入技术在恶性气道疾病中应用 [博士]: 重庆医科大学; 2022.  66. 陈娟, 梁龙, 姚文秀. 晚期NSCLC患者外周血炎性标记物与EGFR-TKIs靶向治疗相关预后的关联及研究进展. *肿瘤预防与治疗* 2022; **35**(12): 1117-25.  67. 石子宜. 外周血NLR、SII对接受PD-1/PD-L1抑制剂治疗的晚期NSCLC患者的疗效及预后价值 [硕士]: 安徽医科大学; 2022.  68. 王田田. 非小细胞肺癌预后影响因素分析及预测模型的建立 [硕士]: 承德医学院; 2022.  69. 谢剑华, 刘苗苗, 彭丽丽, 张荣三, 张洪珍. 系统免疫炎症营养指数与非手术治疗老年非小细胞肺癌患者预后的关系研究. *中国全科医学* 2022; **25**(17): 2082-9.  70. Banna GL, Friedlaender A, Tagliamento M, et al. Biological Rationale for Peripheral Blood Cell-Derived Inflammatory Indices and Related Prognostic Scores in Patients with Advanced Non-Small-Cell Lung Cancer. *Current oncology reports* 2022; **24**(12): 1851-62.  71. Banna GL, Friedlaender A, Tagliamento M, et al. Biological Rationale for Peripheral Blood Cell-Derived Inflammatory Indices and Related Prognostic Scores in Patients with Advanced Non-Small-Cell Lung Cancer. *Current oncology reports* 2022; **24**(12): 1851-62  72. Holtzman L, Moskovitz M, Urban D, et al. dNLR-Based Score Predicting Overall Survival Benefit for The Addition of Platinum-Based Chemotherapy to Pembrolizumab in Advanced NSCLC With PD-L1 Tumor Proportion Score >= 50%. *Clinical lung cancer* 2022; **23**(2): 122-34.  73. Huiru X, Huijing F, Weihong Z, et al. Prediction of immune-related adverse events in non-small cell lung cancer patients treated with immune checkpoint inhibitors based on clinical and hematological markers: Real-world evidence. *Experimental cell research* 2022; **416**(1).  74. Kang MJ, Won YJ, Lee JJ, et al. Cancer Statistics in Korea: Incidence, Mortality, Survival, and Prevalence in 2019. *Cancer research and treatment* 2022; **54**(2): 330-44.  75. L dCW, Danielle G, Abiodun O, et al. Patterns of venous thromboembolism risk, treatment, and outcomes among patients with cancer from uninsured and vulnerable populations. *American journal of hematology* 2022; **97**(8).  76. Huiru X, Huijing F, Weihong Z, et al. Prediction of immune-related adverse events in non-small cell lung cancer patients treated with immune checkpoint inhibitors based on clinical and hematological markers: Real-world evidence. *Experimental cell research* 2022; **416**(1).  77. Liu N, Mao J, Tao P, Chi H, Jia W, Dong C. The relationship between NLR/PLR/LMR levels and survival prognosis in patients with non-small cell lung carcinoma treated with immune checkpoint inhibitors. *Medicine* 2022; **101**(3): e28617.  78. Luigi BG, Alex F, Marco T, et al. Biological Rationale for Peripheral Blood Cell-Derived Inflammatory Indices and Related Prognostic Scores in Patients with Advanced Non-Small-Cell Lung Cancer. *Current oncology reports* 2022; **24**(12).  79. Qiyu F, Jia Y, Wei L, et al. Prognostic Value of Inflammatory and Nutritional Indexes among Advanced NSCLC Patients Receiving PD-1 Inhibitor Therapy. *Clinical and experimental pharmacology & physiology* 2022.  80. Taiki H, Alexis N, Masato K, et al. Cancer Cachexia among Patients with Advanced Non-Small-Cell Lung Cancer on Immunotherapy: An Observational Study with Exploratory Gut Microbiota Analysis. *Cancers* 2022; **14**(21).  81. Tian B-W, Yang Y-F, Yang C-C, et al. Systemic immune-inflammation index predicts prognosis of cancer immunotherapy: systemic review and meta-analysis. *Immunotherapy* 2022; **14**(18): 1481-96.  82. Wan L, Wu C, Luo S, Xie X. Prognostic Value of Lymphocyte-to-Monocyte Ratio (LMR) in Cancer Patients Undergoing Immune Checkpoint Inhibitors. *Disease markers* 2022; **2022**: 3610038.  83. Xia C, Dong X, Li H, et al. Cancer statistics in China and United States, 2022: profiles, trends, and determinants. *Chinese medical journal* 2022; **135**(5): 584-90.  84. Yilmaz M, Baran A, Yilmaz MK. Predictive significance of inflammatory indexes in metastatic nonsmall cell lung cancer patients treated with platinum-doublet chemotherapy. *Journal of cancer research and therapeutics* 2022; **18**(1): 220-3.  85. Yuting P, Hanyan S, Ru J, et al. Analysis of a Systemic Inflammatory Biomarker in Advanced Bile Tract Carcinoma Treated with Anti-PD-1 Therapy: Prognostic and Predictive Significance of Lung Immune Prognostic Index Score. *Journal of Oncology* 2022; **2022**.  86. Zhou Y, Dai M, Zhang Z. Prognostic Significance of the Systemic Immune-Inflammation Index (SII) in Patients With Small Cell Lung Cancer: A Meta-Analysis. *Frontiers in oncology* 2022; **12**: 814727.  87. Ang L, B MS, Jennifer L, et al. Venous thromboembolism risk in cancer patients receiving first-line immune checkpoint inhibitor versus chemotherapy. *American journal of hematology* 2023.  88. D WC, A AM, Lin G, Vishal V, Ashlyn P, Michael K. Health outcomes and healthcare resource utilization among Veterans with stage IV non-small cell lung cancer treated with second-line chemotherapy versus immunotherapy. *PloS one* 2023; **18**(2).  89. Fan R, Chen Y, Xu G, Pan W, Lv Y, Zhang Z. Combined systemic immune-inflammatory index and prognostic nutritional index predict outcomes in advanced non-small cell lung cancer patients receiving platinum-doublet chemotherapy. *Frontiers in oncology* 2023; **13**.  90. Himuro H, Nakahara Y, Igarashi Y, et al. Clinical roles of soluble PD-1 and PD-L1 in plasma of NSCLC patients treated with immune checkpoint inhibitors. *Cancer immunology, immunotherapy : CII* 2023.  91. Jenkins R, Walker J, Roy UB. 2022 cancer statistics: Focus on lung cancer. *Future oncology (London, England)* 2023.  92. Ju W, Zheng R, Zhang S, et al. Cancer statistics in Chinese older people, 2022: current burden, time trends, and comparisons with the US, Japan, and the Republic of Korea. *Science China Life sciences* 2023; **66**(5): 1079-91.  93. Mahiat C, Bihin B, Duplaquet F, et al. Systemic Inflammation/Nutritional Status Scores Are Prognostic but Not Predictive in Metastatic Non-Small-Cell Lung Cancer Treated with First-Line Immune Checkpoint Inhibitors. *International journal of molecular sciences* 2023; **24**(4).  94. Ruiyun F, Ying C, Guopeng X, Wen P, Yantian L, Zhongwei Z. Combined systemic immune-inflammatory index and prognostic nutritional index predict outcomes in advanced non-small cell lung cancer patients receiving platinum-doublet chemotherapy. *Frontiers in oncology* 2023; **13**.  95. Sakai A, Iijima H, Ebisumoto K, et al. Prognostic Value of Inflammatory and Nutritional Biomarkers of Immune Checkpoint Inhibitor Treatment for Recurrent or Metastatic Squamous Cell Carcinoma of the Head and Neck. *Cancers* 2023; **15**(7).  96. Siegel RL, Miller KD, Wagle NS, Jemal A. Cancer statistics, 2023. *CA: a cancer journal for clinicians* 2023; **73**(1): 17-48.  97. Zheng RS, Zhang SW, Sun KX, et al. [Cancer statistics in China, 2016]. *Zhonghua zhong liu za zhi [Chinese journal of oncology]* 2023; **45**(3): 212-20.  98. Banna GL, Friedlaender A, Tagliamento M, et al. Biological Rationale for Peripheral Blood Cell-Derived Inflammatory Indices and Related Prognostic Scores in Patients with Advanced Non-Small-Cell Lung Cancer. *Current oncology reports* 2022; **24**(12): 1851-62.  99. Bauckneht M, Genova C, Rossi G, et al. The Role of the Immune Metabolic Prognostic Index in Patients with Non-Small Cell Lung Cancer (NSCLC) in Radiological Progression during Treatment with Nivolumab. *Cancers* 2021; **13**(13).  100. Bauckneht M, Genova C, Rossi G, et al. The Role of the Immune Metabolic Prognostic Index in Patients with Non-Small Cell Lung Cancer (NSCLC) in Radiological Progression during Treatment with Nivolumab. *Cancers* 2021; **13**(13).  101. Cédric M, Benoît B, Fabrice D, et al. Systemic Inflammation/Nutritional Status Scores Are Prognostic but Not Predictive in Metastatic Non-Small-Cell Lung Cancer Treated with First-Line Immune Checkpoint Inhibitors. *International journal of molecular sciences* 2023; **24**(4).  102. Chongwu L, Junqi W, Long J, et al. The predictive value of inflammatory biomarkers for major pathological response in non-small cell lung cancer patients receiving neoadjuvant chemoimmunotherapy and its association with the immune-related tumor microenvironment: a multi-center study. *Cancer immunology, immunotherapy : CII* 2022; **72**(3).  103. Claire G, Pascaline BR, Anne J, et al. Predictive and prognostic value of systemic inflammatory response biomarkers in patients receiving nivolumab for metastatic non-small cell lung cancer (NSCLC). *Journal of Clinical Oncology* 2017; **35**(15_suppl).  104. Colum D, Eileen M, Gerard PD, et al. Clinical haematological biomarkers: Derived neutrophil-to-lymphocyte ratio (dNLR), platelet-to-lymphocyte ratio (PLR), and prognostic nutritional index (PNI) and their relationship to survival outcomes in non small cell lung cancer (NSCLC) treated with immunotherapy: A multicenter review. *Journal of Clinical Oncology* 2019; **37**(15_suppl).  105. Elena RS, Arsela P, Alex F, et al. Prognostic scores including peripheral blood-derived inflammatory indices in patients with advanced non-small-cell lung cancer treated with immune checkpoint inhibitors. *Critical reviews in oncology/hematology* 2022; **179**.  106. Fan R, Chen Y, Xu G, Pan W, Lv Y, Zhang Z. Combined systemic immune-inflammatory index and prognostic nutritional index predict outcomes in advanced non-small cell lung cancer patients receiving platinum-doublet chemotherapy. *Frontiers in oncology* 2023; **13**: 996312.  107. Fang Q, Yu J, Li W, et al. Prognostic value of inflammatory and nutritional indexes among advanced NSCLC patients receiving PD-1 inhibitor therapy. *Clinical and experimental pharmacology & physiology* 2023; **50**(2): 178-90.  108. G M, E S, K S, et al. Association of the advanced lung cancer inflammation index (ALI) with immune checkpoint inhibitor efficacy in patients with advanced non-small-cell lung cancer. *ESMO open* 2021; **6**(5).  109. Ito K, Hashimoto K, Kaira K, et al. Clinical impact of inflammatory and nutrition index based on metabolic tumor activity in non‑small cell lung cancer treated with immunotherapy. *Oncol Lett* 2024; **27**(3): 110.  110. Ito K, Hashimoto K, Kaira K, et al. Clinical impact of inflammatory and nutrition index based on metabolic tumor activity in non‑small cell lung cancer treated with immunotherapy. *Oncol Lett* 2024; **27**(3): 110.  111. L. BG, Ornella C, Sethupathi M, et al. Efficacy outcomes and prognostic factors from real-world patients with advanced non-small-cell lung cancer treated with first-line chemoimmunotherapy: The Spinnaker retrospective study. *International Immunopharmacology* 2022; **110**.  112. Alessandro R, Antonino S, Maria P, et al. Association between baseline absolute neutrophil count (ANC), derived neutrophil-to-lymphocyte ratio (dNLR), and platelet-to-lymphocyte ratio (PLR) and response to nivolumab (Nivo) in non-small cell lung cancer (NSCLC): A preliminary analysis. *Journal of Clinical Oncology* 2017; **35**(15_suppl).  113. Peng F, Hu D, Lin X, et al. The monocyte to red blood cell count ratio is a strong predictor of postoperative survival in colorectal cancer patients: The Fujian prospective investigation of cancer (FIESTA) study. *J Cancer* 2017; **8**(6): 967-75.  114. Nakamura K, Smyth MJ. Targeting cancer-related inflammation in the era of immunotherapy. *Immunology and cell biology* 2017; **95**(4): 325-32.  115. Nakamura K, Smyth MJ. Targeting cancer-related inflammation in the era of immunotherapy. *Immunology and cell biology* 2017; **95**(4): 325-32.  116. Liu J, Li S, Zhang S, et al. Systemic immune-inflammation index, neutrophil-to-lymphocyte ratio, platelet-to-lymphocyte ratio can predict clinical outcomes in patients with metastatic non-small-cell lung cancer treated with nivolumab. *Journal of clinical laboratory analysis* 2019; **33**(8): e22964.  117. Peng F, Hu D, Lin X, et al. The monocyte to red blood cell count ratio is a strong predictor of postoperative survival in colorectal cancer patients: The Fujian prospective investigation of cancer (FIESTA) study. *J Cancer* 2017; **8**(6): 967-75.  118. Rubio XM, Rueda AG, Antoñanzas M, et al. Applicability of lung immune prognostic index (LIPI) to predict efficacy of first-line pembrolizumab in advanced non-small cell lung cancer (NSCLC). *Annals of Oncology* 2019; **30**(Supl.5).  119. Rubio XM, Rueda AG, Antoñanzas M, et al. Applicability of lung immune prognostic index (LIPI) to predict efficacy of first-line pembrolizumab in advanced non-small cell lung cancer (NSCLC). *Annals of Oncology* 2019; **30**(Supl.5).  120. 郭美莹. 智能影像组学在预测非小细胞肺癌免疫治疗疗效及放射性肺炎中的研究 [博士]: 山东大学; 2020.  121. Rubio XM, Rueda AG, Antoñanzas M, et al. Applicability of lung immune prognostic index (LIPI) to predict efficacy of first-line pembrolizumab in advanced non-small cell lung cancer (NSCLC). *Annals of Oncology* 2019; **30**(Supl.5).  122. Matsubara T, Takamori S, Haratake N, et al. The impact of immune-inflammation-nutritional parameters on the prognosis of non-small cell lung cancer patients treated with atezolizumab. *Journal of thoracic disease* 2020; **12**(4): 1520-8.  123. Matsubara T, Takamori S, Haratake N, et al. The impact of immune-inflammation-nutritional parameters on the prognosis of non-small cell lung cancer patients treated with atezolizumab. *Journal of thoracic disease* 2020; **12**(4): 1520-8.  124. 石子宜, 郝吉庆. 外周血炎性标志物与PD-1/PD-L1抑制剂治疗肺癌疗效及预后的相关性. *临床肺科杂志* 2022; **27**(10): 1527-32+38.  125. 吴敏. 晚期NSCLC不同转移灶放疗对机体免疫系统激活及免疫治疗疗效影响差异 [硕士]: 山东大学; 2022.  126. Qi WX, Xiang Y, Zhao S, Chen J. Assessment of systematic inflammatory and nutritional indexes in extensive-stage small-cell lung cancer treated with first-line chemotherapy and atezolizumab. *Cancer immunology, immunotherapy : CII* 2021; **70**(11): 3199-206.  127. Holtzman L, Moskovitz M, Urban D, et al. dNLR-Based Score Predicting Overall Survival Benefit for The Addition of Platinum-Based Chemotherapy to Pembrolizumab in Advanced NSCLC With PD-L1 Tumor Proportion Score ≥50. *Clinical lung cancer* 2022; **23**(2): 122-34.  128. Holtzman L, Moskovitz M, Urban D, et al. dNLR-Based Score Predicting Overall Survival Benefit for The Addition of Platinum-Based Chemotherapy to Pembrolizumab in Advanced NSCLC With PD-L1 Tumor Proportion Score ≥50. *Clinical lung cancer* 2022; **23**(2): 122-34.  129. 邓隽军, 赵大勇, 李淼. 免疫检查点抑制剂在非小细胞肺癌治疗中的不良反应及危险因素. *国际肿瘤学杂志* 2023; **50**(09): 564-8.  130. 段惠娟, 李希, 党傲, 赖雪梅, 张家春. 系统免疫炎症指数与外周血相关指标对进展期非小细胞肺癌患者预后的预测价值. *实用医院临床杂志* 2023; **20**(05): 74-8.  131. 石明伟, 王俊康, 王静. 系统免疫炎症营养指数对接受免疫检查点抑制剂治疗的非小细胞肺癌患者临床疗效及预后的评估价值研究. *解放军医学院学报* 2023; **44**(12): 1372-8+83.  132. 谢斌, 韦文萍, 徐秋燕, 韩传军. 系统免疫炎症指数联合循环肿瘤细胞评价老年非小细胞肺癌PD-1抑制剂治疗反应. *中国老年学杂志* 2023; **43**(11): 2604-6.  133. A. BE, Hsiang CW, Sunil M, B. KR, Jun Z, H. BE. Treatment at Twilight: An Analysis of Therapy Patterns and Outcomes in Adults 80 Years and Older With Advanced or Metastatic NSCLC. *JTO Clinical and Research Reports* 2023; **4**(10).  134. Alessio R, Ornella C, Andrea M, et al. Assessing the role of colonic and other anatomical sites uptake by [18 F]FDG-PET/CT and immune-inflammatory peripheral blood indexes in patients with advanced non-small cell lung cancer treated with first-line immune checkpoint inhibitors. *Thoracic cancer* 2023; **14**(24).  135. Anpalakhan S, Signori A, Cortellini A, et al. Using peripheral immune-inflammatory blood markers in tumors treated with immune checkpoint inhibitors: An INVIDIa-2 study sub-analysis. *iScience* 2023; **26**(11): 107970.  136. Fuhao X, He Z, Dali X, et al. Tertiary lymphoid structures combined with biomarkers of inflammation are associated with the efficacy of neoadjuvant immunochemotherapy in resectable non-small cell lung cancer: A retrospective study. *Thoracic cancer* 2023; **15**(2).  137. He G, Wang J, Feng M. Predictive value of inflammatory markers of peripheral blood cells on prognosis in the advanced non-small cell lung cancer with immune therapy. *J Int Oncol* 2023; **50**(06): 321-7.  138. Anpalakhan S, Signori A, Cortellini A, et al. Using peripheral immune-inflammatory blood markers in tumors treated with immune checkpoint inhibitors: An INVIDIa-2 study sub-analysis. *iScience* 2023; **26**(11): 107970.  139. K. T, R. B, M. G, K. K, E. P. Determining the Optimal Sequence of Immunotherapy and Lymph Node Irradiation among Patients with Cancer: A Propensity Score Matched Analysis. *International Journal of Radiation Oncology, Biology, Physics* 2023; **117**(2S).  140. K. T, R. B, M. G, K. K, E. P. Determining the Optimal Sequence of Immunotherapy and Lymph Node Irradiation among Patients with Cancer: A Propensity Score Matched Analysis. *International Journal of Radiation Oncology, Biology, Physics* 2023; **117**(2S).  141. Liu C, Zhao H, Zhang R, Guo Z, Wang P, Qu Z. Prognostic value of nutritional and inflammatory markers in patients with hepatocellular carcinoma who receive immune checkpoint inhibitors. *Oncol Lett* 2023; **26**(4): 437.  142. Liu C, Zhao H, Zhang R, Guo Z, Wang P, Qu Z. Prognostic value of nutritional and inflammatory markers in patients with hepatocellular carcinoma who receive immune checkpoint inhibitors. *Oncol Lett* 2023; **26**(4): 437.  143. Qilin H, Chenyu L, Peng S, et al. Peripheral blood inflammatory biomarkers dynamics reflect treatment response and predict prognosis in non-small cell lung cancer patients with neoadjuvant immunotherapy. *Cancer science* 2023; **114**(12).  144. Rizzo A, Cantale O, Mogavero A, et al. Assessing the role of colonic and other anatomical sites uptake by [(18) F]FDG-PET/CT and immune-inflammatory peripheral blood indexes in patients with advanced non-small cell lung cancer treated with first-line immune checkpoint inhibitors. *Thorac Cancer* 2023; **14**(24): 2473-83.  145. Wang J. 血液炎症复合指标与非小细胞肺癌患者免疫治疗预后的相关性研究 [硕士]: 山西医科大学; 2023.  146. Zheng F, Meng Q, Zhang L, et al. Prognostic roles of hematological indicators for the efficacy and prognosis of immune checkpoint inhibitors in patients with advanced tumors: a retrospective cohort study. *World J Surg Oncol* 2023; **21**(1): 198.  147. 毕周奎. 慢性阻塞性肺疾病及其外周血炎症指标对晚期非小细胞肺癌免疫治疗疗效的影响 [硕士]: 中国人民解放军陆军军医大学; 2024.  148. 吕浩. CHIT1作为免疫治疗生物标志物的初步探究 [硕士]: 中国人民解放军陆军军医大学; 2024.  149. Asano Y, Hayashi K, Takeuchi A, et al. Combining dynamics of serum inflammatory and nutritional indicators as novel biomarkers in immune checkpoint inhibitor treatment of non-small-cell lung cancer with bone metastases. *International immunopharmacology* 2024; **136**.  150. Baek JM, Cha H, Moon Y, et al. A Systemic Immune Inflammation Index and PD-L1 (SP142) Expression as a Potential Combined Biomarker of the Clinical Benefit of Chemo-Immunotherapy in Extensive-Stage Small-Cell Lung Cancer. *J Clin Med* 2024; **13**(5).  151. Bi ZK, Xu Y, Guo L, et al. Effect of peripheral blood inflammatory indicators on the efficacy of immunotherapy in patients with advanced non-small cell lung cancer and chronic obstructive pulmonary disease. *Zhonghua yi xue za zhi* 2024; **104**(18).  152. Bi ZK, Xu Y, Guo L, et al. Effect of peripheral blood inflammatory indicators on the efficacy of immunotherapy in patients with advanced non-small cell lung cancer and chronic obstructive pulmonary disease. *Zhonghua yi xue za zhi* 2024; **104**(18).  153. Deng C, Liao J, Fu Z, et al. Systemic immune index predicts tumor-infiltrating lymphocyte intensity and immunotherapy response in small cell lung cancer. *Translational lung cancer research* 2024; **13**(2).  154. Guigay J, Ortholan C, Vansteene D, et al. Cetuximab versus methotrexate in first-line treatment of older, frail patients with inoperable recurrent or metastatic head and neck cancer (ELAN UNFIT): a randomised, open-label, phase 3 trial. *The lancet Healthy longevity* 2024; **5**(3).  155. He J, Liang G, Yu H, Lin C, Shen W. Evaluating the predictive significance of systemic immune-inflammatory index and tumor markers in lung cancer patients with bone metastases. *Frontiers in oncology* 2024; **13**.  156. Hua Q. Predictive value of peripheral hematological indicators in first-line immunotherapy for extensive small-cell lung cancer patients [Master]: Ganlan Medical Colloge; 2024.  157. Ito K, Hashimoto K, Kaira K, et al. Clinical impact of inflammatory and nutrition index based on metabolic tumor activity in non‑small cell lung cancer treated with immunotherapy. *Oncology letters* 2024; **27**(3).  158. Kim YJ, Lee M, Kim EH, et al. Real-world Incidences and Risk Factors of Immune-related Adverse Events in Patients Treated with Immune Checkpoint Inhibitors: A Nationwide Retrospective Cohort Study. *Cancer letters* 2024; **596**.  159. Li Y. 系统免疫炎症指数及淋巴细胞亚群对于肺癌免疫治疗疗效及预后研究 [硕士]: 吉林大学; 2024.  160. Roller A, Davydov II, Schwalie PC, et al. Tumor-agnostic transcriptome-based classifier identifies spatial infiltration patterns of CD8+T cells in the tumor microenvironment and predicts clinical outcome in early-phase and late-phase clinical trials. *Journal for immunotherapy of cancer* 2024; **12**(4).  161. Shao H, Zhu J, Shi L, et al. Value of computed tomography radiomics combined with inflammation indices in predicting the efficacy of immunotherapy in patients with locally advanced and metastatic non-small cell lung cancer. *Journal of thoracic disease* 2024; **16**(5): 3213-27.  162. Tang L. Application of peripheral blood markers to predict immune-related toxicity and prognosis in patients with advanced non-small cell lung cancer [Master]: Dalian Medical University; 2024.  163. Xiang Y, Chen L. The clinical value of the systemic immune-inflammation index for major pathological response in non-small cell lung cancer patients receiving neoadjuvant chemoimmunotherapy. *Transactions on Cancer* 2024; **5**(1).  164. Xu F, Zhu H, Xiong D, et al. Tertiary lymphoid structures combined with biomarkers of inflammation are associated with the efficacy of neoadjuvant immunochemotherapy in resectable non-small cell lung cancer: A retrospective study. *Thorac Cancer* 2024; **15**(2): 172-81.  165. Xu F, Zhu H, Xiong D, et al. Tertiary lymphoid structures combined with biomarkers of inflammation are associated with the efficacy of neoadjuvant immunochemotherapy in resectable non-small cell lung cancer: A retrospective study. *Thorac Cancer* 2024; **15**(2): 172-81.  166. Liu J, Li S, Zhang S, et al. Systemic immune-inflammation index, neutrophil-to-lymphocyte ratio, platelet-to-lymphocyte ratio can predict clinical outcomes in patients with metastatic non-small-cell lung cancer treated with nivolumab. *Journal of clinical laboratory analysis* 2019; **33**(8).  167. Qi X, Ziwei H, Lingli X, et al. Post-treatment neutrophil-to-lymphocyte ratio (NLR) predicts response to anti-PD-1/PD-L1 antibody in SCLC patients at early phase. *Cancer immunology, immunotherapy : CII* 2020; **70**(prepublish).  168. Romain-David S, Jean-Baptiste A, Etienne G-L, et al. Prognostic value of inflammatory response biomarkers using peripheral blood and [18F]-FDG PET/CT in advanced NSCLC patients treated with first-line chemo- or immunotherapy. *Lung Cancer* 2021; **159**.  169. Wei X, Zhang C, Zang F, Chen P. Preliminary study on inflammatory markers for predicting the efficacy and prognosis of anti-PD-1 antibody treatment in patients with non-small cell lung cancer. *Chin J Clin Oncol* 2021; **48**(11): 547-52.  170. Yang X. The predictive role of hematological indicators in the efficacy of immune checkpoint inhibitors in patients with NSCLC [Master]: Jilin University; 2021.  171. Yi X. Study on the efficacy and prognostic predictive value of systemic immune-inflammation index and prognostic nutrition index in patients with advanced non-small celllung cancer treated with PD-1 inhibitors [Master]: Nanchang University; 2021.  172. Banna GL, Cantale O, Muthuramalingam S, et al. Efficacy outcomes and prognostic factors from real-world patients with advanced non-small-cell lung cancer treated with first-line chemoimmunotherapy: The Spinnaker retrospective study. *International Immunopharmacology* 2022; **110**.  173. Hu J. PD-L1 expression and hematological markers in the prediction of immunotherapy response and prognosis in locally advanced and metastatic non-small cell lung cancer patients [Master]: China Medical University; 2022.  174. Liu J, Li M. Predictive value of systemic immune inflammation index in the treatment of PD ⁃1/PD ⁃L1 immune checkpoint inhibitors in non⁃small cell lung cancer. *The Journal of Practical Medicine* 2022; **38**(07): 904-8.  175. Xu X. The prognostic value of peripheral blood inflammatory markers in patients with advanced non-small cell lung cancer treated with anti-PD-1 immunotherapy [Master]: Jilin University; 2022.  176. Fang Q, Yu J, Li W, et al. Prognostic value of inflammatory and nutritional indexes among advanced NSCLC patients receiving PD-1 inhibitor therapy. *Clinical and Experimental Pharmacology and Physiology* 2023; **50**(2): 178-90.  177. Yamaguchi O, Kaira K, Imai H, et al. Clinical Utility of Inflammatory and Nutritious Index as Therapeutic Prediction of Nivolumab plus Ipilimumab in Advanced Non-Small Cell Lung Cancer. *Oncology* 2024; **102**(3): 271-82.  178. 刘成成. SII/ALB比值预测免疫检查点抑制剂治疗恶性肿瘤疗效真实世界研究 [硕士]: 大连医科大学; 2021.  179. 滕华, 刘苗苗, 张荣三, 谢剑华, 张洪珍. 系统免疫炎症指数对小细胞肺癌患者预后的影响. *河北医科大学学报* 2021; **42**(08): 886-90. | ☆  ☆  Duplicated record  ☆  ☆  ☆  ☆  ☆  ☆  ☆  ☆  ☆  Duplicated record  ☆  ☆  ☆  ☆  ☆  ☆  ☆  Duplicated record  ☆  ☆  Duplicated record  ☆  ☆  ☆  ☆  ☆  ☆  ☆  ☆  ☆  ☆  Duplicated record  ☆  Duplicated record  ☆  Duplicated record  ☆  ☆  Duplicated record  Duplicated record  ☆  ☆  ☆  ☆  ☆  ☆  ☆  ☆  Duplicated record  ☆  Duplicated record  Duplicated record  ☆  ☆  ☆  ☆  Included  ☆  Duplicated record  Included  ☆  ☆  ☆  ☆  ☆  ☆  ☆  Duplicated record  ☆  ☆  ☆  ☆  ☆  Duplicated record  ☆  ☆  Duplicated record  ☆  ☆  ☆  ☆  ☆  ☆  ☆  ☆  ☆  Duplicated record  ☆  ☆  ☆  Excluded due to insufficient data  ☆  ☆  ☆  ☆  ☆  Duplicated record  ☆  ☆  ☆  ☆  ☆  Excluded after reviewing the abstract  ☆  Included  ☆  Excluded due to insufficient data  Duplicated record  Duplicated record  ☆  ☆  Duplicated record  Duplicated record  Included  Duplicated record  Excluded after reviewing the abstract  Duplicated record  ☆  Duplicated record  Duplicated record  ☆  ☆  Excluded after reviewing the abstract  Duplicated record  Included  Duplicated record  ☆  Excluded due to insufficient data  Excluded after reviewing the abstract  Excluded due to insufficient data  ☆  Included  ☆  Excluded due to insufficient data  Included  Excluded  Duplicated record  ☆  Duplicated record  ☆  Excluded due to insufficient data  ☆  Excluded after reviewing the abstract  ☆  Excluded after reviewing the abstract  ☆  ☆  Included  Included  Duplicated record  Excluded after reviewing the abstract  ☆  Excluded after reviewing the abstract  Included  Excluded after reviewing the abstract  ☆  Excluded due to duplicated data  Excluded due to insufficient data  Included  Excluded after reviewing the abstract  Duplicated record  ☆  Duplicated record  Duplicated record  Excluded after reviewing the abstract  Included  Included  Included  Included  Included  Included  Included  Excluded after reviewing the abstract  Included  Duplicated record  Excluded after reviewing the abstract |

☆: Excluded after reviewing the title.
