# Supplementary material for: Prognostic value of the systemic immune-inflammation index in lung cancer patients receiving immune checkpoint inhibitors: A meta-analysis
Source: PLoS One. 2024 Nov 1;19(11):e0312605. doi: 10.1371/journal.pone.0312605 (PMC11530041; doi:10.1371/journal.pone.0312605)
Supplement: Attachment — Submitted filename: answer.docx [file pone.0312605.s003.docx]

**Response to issues by editor:**

**Question 1:** Grant information and Financial Disclosure

**Answer 1:** We have changed the grand information as follows: “Funding: None”.

**Question 2:** Data sharing statement

**Answer 2:** This is a meta-analysis, all the data used have been presented in the manuscript. Therefore, the sharing for raw data is not applicable in our manuscript.

**Question 3:** PRISMA checklist

**Answer 3:** The checklist has been uploaded.

**Response to editor:**

**Question 1.** When submitting your revision, we need you to address these additional requirements.

Please ensure that your manuscript meets PLOS ONE's style requirements, including those for file naming. The PLOS ONE style templates can be found at

https://journals.plos.org/plosone/s/file?id=wjVg/PLOSOne_formatting_sample_main_body.pdf and

https://journals.plos.org/plosone/s/file?id=ba62/PLOSOne_formatting_sample_title_authors_affiliations.pdf.

**Answer 1:** Thanks for your comment. We have carefully modified our manuscript according to the guidelines as request.

**Question 2**. We noticed you have some minor occurrence of overlapping text with the following previous publication(s), which needs to be addressed:

10.7759/cureus.52720

In your revision ensure you cite all your sources (including your own works), and quote or rephrase any duplicated text outside the methods section. Further consideration is dependent on these concerns being addressed.

**Answer 2:** We have carefully modified our manuscript to reduce the overlapping text.

**Question 3.** We note that the grant information you provided in the ‘Funding Information’ and ‘Financial Disclosure’ sections do not match.

When you resubmit, please ensure that you provide the correct grant numbers for the awards you received for your study in the ‘Funding Information’ section.

**Answer 3:** We have updated the funding information and financial disclosure part in the submission system.

**Response to Reviewer #1:**

Thanks for giving me the opportunity to review this interesting work. In overall, this manuscript was well designed and organized. I have only a few minor concerns about the work.

**Question 1**. Some grammar and spelling mistakes could be found in the manuscript.

**Answer 1:** This manuscript has been edited by AJE service with the verification code 5FF8-A3F4-9204-0BA6-ACEB.

**Question 2.** This work is a meta-analysis. In general, the study design should be noted in the title.

**Answer 2:** We have revised the title to “**Prognostic value of systemic immune-inflammation index in lung cancer patients receiving immune checkpoint inhibitors: a meta-analysis**”

**Question 3.** Databases searched should be mentioned in the abstract.

**Answer 3:** Databases searched has been added in the abstract. “PubMed, EMBASE, Web of Science, and CNKI databases”.

**Question 4.** Although the authors indicated in the inclusion criteria that SII was calculated before immunotherapy, I would suggest the authors deleting the term "pretreatment" which might lead to ambiguity. Patients may receive other therapies before the immunotherapy?

**Answer 4:** Thanks for your question. After careful consideration, we have deleted the term of “pretreatment”.

**Response to Reviewer #2:** The authors eplored the prognostic role of SII in ICIs treated patients. Congratulations for this successful work. In overall, this study was well designed and written. I have several minor concerns about this paper.

**Question 1.** Study design type should be specificly described in the title.

**Answer 1:** We have revised the title to “**Prognostic value of systemic immune-inflammation index in lung cancer patients receiving immune checkpoint inhibitors: a meta-analysis**”

**Question 2.** "patients were divided into elevated and normal-SII groups according" This sentence is not correct?

**Answer 2:** This sentence has been modified.

**Question 3.** The results of quality assessment should be also described in the results part.

**Answer 3:** We have added the description about the quality assessment results. “All studies were with a NOS score≥6.”

**Question 4.** Besides, the authors should carefully check and revise the manuscript for grammar or spelling errors.

**Answer 4:** This manuscript has been edited by AJE service with the verification code 5FF8-A3F4-9204-0BA6-ACEB.
